# The tarsal taste of honey bees: behavioral and electrophysiological analyses

**DOI:** 10.3389/fnbeh.2014.00025

**Published:** 2014-02-04

**Authors:** Maria Gabriela de Brito Sanchez, Esther Lorenzo, Songkun Su, Fanglin Liu, Yi Zhan, Martin Giurfa

**Affiliations:** ^1^Centre National de la Recherche Scientifique (CNRS), Research Center on Animal Cognition (UMR5169)Toulouse, France; ^2^University Paul-Sabatier, Research Center on Animal Cognition (UMR5169)Toulouse, France; ^3^College of Animal Sciences, Zhejiang UniversityHangzhou, China; ^4^Xishuangbanna Tropical Botanical Garden, Chinese Academy of SciencesKunming, China

**Keywords:** taste, gustation, gustatory receptors, insect, honey bee, tarsi, proboscis extension reflex, electrophysiology

## Abstract

Taste plays a crucial role in the life of honey bees as their survival depends on the collection and intake of nectar and pollen, and other natural products. Here we studied the tarsal taste of honey bees through a series of behavioral and electrophysiological analyses. We characterized responsiveness to various sweet, salty and bitter tastants delivered to gustatory sensilla of the fore tarsi. Behavioral experiments showed that stimulation of opposite fore tarsi with sucrose and bitter substances or water yielded different outcomes depending on the stimulation sequence. When sucrose was applied first, thereby eliciting proboscis extension, no bitter substance could induce proboscis retraction, thus suggesting that the primacy of sucrose stimulation induced a central excitatory state. When bitter substances or water were applied first, sucrose stimulation could still elicit proboscis extension but to a lower level, thus suggesting central inhibition based on contradictory gustatory input on opposite tarsi. Electrophysiological experiments showed that receptor cells in the gustatory sensilla of the tarsomeres are highly sensitive to saline solutions at low concentrations. No evidence for receptors responding specifically to sucrose or to bitter substances was found in these sensilla. Receptor cells in the gustatory sensilla of the claws are highly sensitive to sucrose. Although bees do not possess dedicated bitter-taste receptors in the tarsi, indirect bitter detection is possible because bitter tastes inhibit sucrose receptor cells of the claws when mixed with sucrose solution. By combining behavioral and electrophysiological approaches, these results provide the first integrative study on tarsal taste detection in the honey bee.

## Introduction

Taste is a fundamental sensory modality for individual survival as it allows discriminating edible from non-edible items, which may cause significant harm or death (Scott, 2004, 2005; Yarmolinsky et al., 2009; Carleton et al., 2010; De Brito Sanchez and Giurfa, 2011). In the definition adopted throughout this work, taste is a specific form of contact chemoreception relying on specialized receptor neurons (gustatory receptor neurons or GRNs) tuned to respond to different kinds of substances in a food-related context. Not all contact chemoreceptors intervene in a food-related context, and are not, therefore, GRNs. For instance, contact chemoreceptors may be tuned to detect pheromone or kairomone compounds.

Insects, in particular the fruit fly *Drosophila melanogaster*, constitute powerful models for the study of the physiological principles of taste perception (Thorne et al., 2004; Hallem et al., 2006; Cobb et al., 2009; Montell, 2009). In insects, GRNs are primary neurons that are contained within cuticular structures called *gustatory or taste sensilla* (Dahanukar et al., 2005; Hallem et al., 2006). These sensilla contain two to four GRNs, one mechanosensory neuron and several types of accessory cells. Gustatory sensilla are not restricted to the region around the mouth but are usually distributed over different regions of the body surface and appendages such as the antennae, mouth parts, leg tarsi, and margins of the wings. Molecular gustatory receptors (Grs) located on the membrane of GRNs confer taste specificity and mediate appropriate responses to tastes (Dahanukar et al., 2005; Vosshall and Stocker, 2007; Cobb et al., 2009; Montell, 2009). In the fruit fly, 68 Grs encoded by 60 genes through alternative splicing have been identified (Clyne et al., 2000; Dunipace et al., 2001; Scott et al., 2001; Robertson et al., 2003). Some Grs have been linked to specific sweet and bitter tastants (Ishimoto and Tanimura, 2004; Thorne et al., 2004; Montell, 2009).

Yet, the sequencing of other insect genomes has shown that the taste organization of the fruit fly is not shared by all insects as different life styles led to modifications of the gustatory repertoire. In the honey bee *Apis mellifera*, which has a model status for research on learning, memory, and perception (Giurfa, 2007; Galizia et al., 2011), only 10 Grs were identified in the genome (The Honeybee Genome Sequencing Consortium, 2006). None of them shares homologies with bitter-tuned Grs of *Drosophila*, a finding that was interpreted as the result of taste specialization on sweet and non-toxic tastants (Robertson and Wanner, 2006). Until now, the specific tastants of these Grs remain unknown.

Behavioral and electrophysiological approaches have been used to characterize taste perception in bees (review in De Brito Sanchez, 2011). Sensitivity to sugars and saline solutions was found at the level of the antennae and mouth parts both in behavioral experiments and in electrophysiological recordings of single sensilla (Whitehead and Larsen, 1976a,b; Whitehead, 1978; Haupt, 2004; De Brito Sanchez et al., 2005). Interestingly, no sensitivity to bitter substances could be detected (De Brito Sanchez et al., 2005; Ayestaran et al., 2010), even if some bitter substances inhibit the response of sucrose GRNs when mixed with sucrose solution (De Brito Sanchez et al., 2005).

Despite their long-claimed role in gustation (Frings and Frings, 1949), the gustatory sensitivity of the honey bee fore-tarsi have remained mostly unexplored (De Brito Sanchez, 2011). Here we provide the first extensive account of honey bee tarsal gustation and characterized behavioral and electrophysiological responses to sweet, bitter, and saline substances at the level of these appendages.

## Materials and methods

Free-flying honey bee foragers (female workers), *Apismellifera*, were caught in the morning of every experimental day upon return to the hive entrance. They were placed in glass vials and cooled in ice until they stopped moving. They were then prepared in the laboratory for behavioral or electrophysiological experiments.

### Behavioral experiments

#### Insects

**Figure 1:**
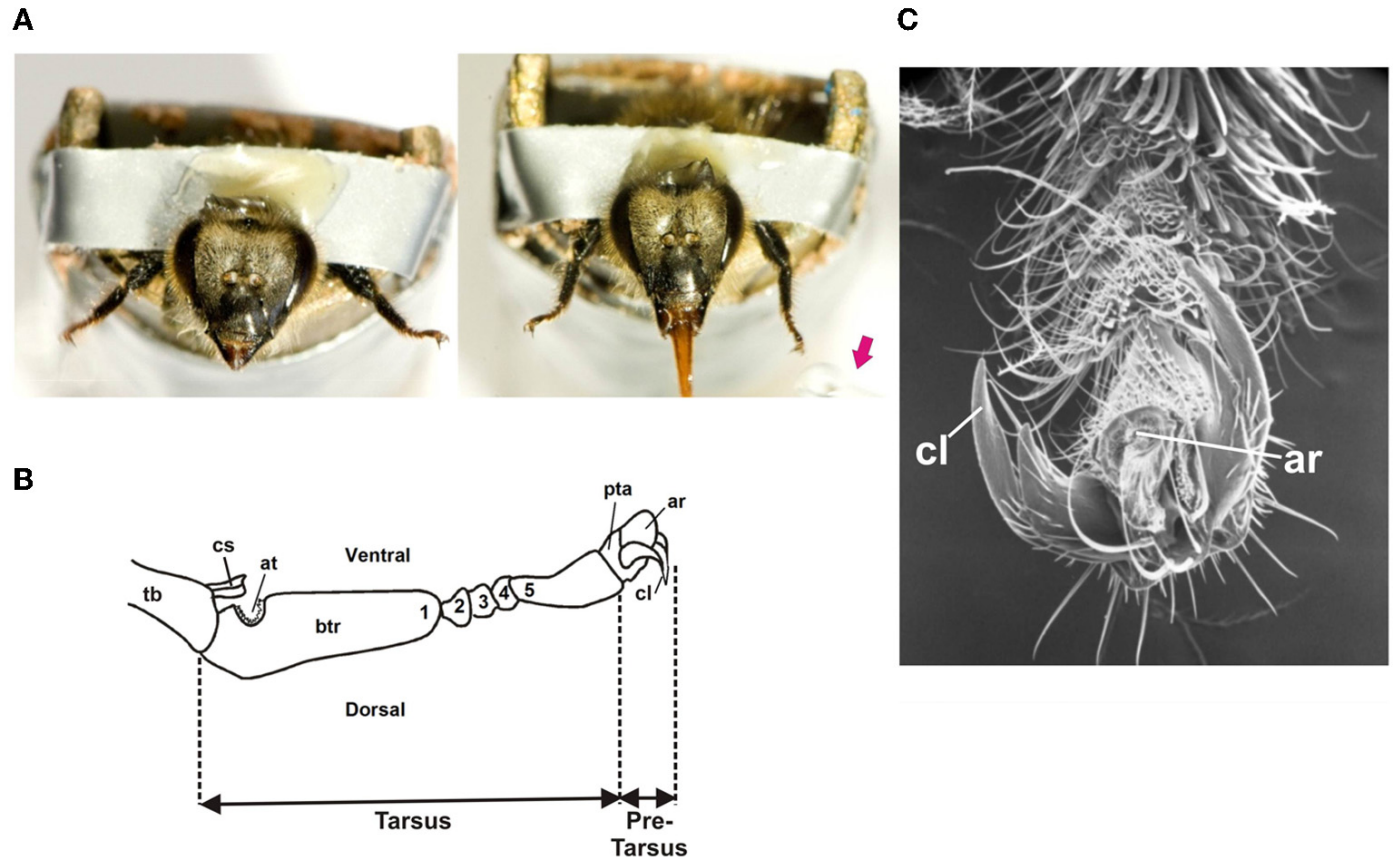
**(A)** Left: Honey bee with amputated antennae harnessed in a metal tube with its forelegs fixed wide open in order to allow tarsal gustatory stimulation. Right: Proboscis extension reflex (PER) upon tarsal stimulation with a drop of sucrose solution (red arrow) delivered to the left tarsus. **(B)** Scheme of the distal segments of a honey bee foreleg showing the tarsus and the pretarsus. The tarsus has five tarsomeres: a basitarsus (btr: 1), which is the largest tarsomere, and 4 smaller tarsomeres (2–5). The basitarsus presents a notch of antenna cleaner (at) and the tibia (Tb) a closing spine (cs). The distally situated pretarsus (pta) bears a pair of lateral bifid claws (cl) and an arolium (ar), a small pad used to increase adhesion. **(C)** Detail of the pretarsus (pta) of a foreleg: cl: claws; ar: arolium.

Bees were mounted individually in small metal tubes from which only their head and fore-tarsi protruded (De Brito Sanchez et al., 2008). The forelegs were fixed wide open in order to facilitate their stimulation (Figure 1A). The insects were kept for 2 h in a dark and humid container before the experiments. The antennae were amputated (Figure 1A) in order to avoid antennal interference in trials in which two gustatory stimuli had to be delivered on left and right fore-tarsi (De Brito Sanchez et al., 2008). Amputation occurred at least 2–3 h before experiments. Both antennae were cut with fine scissors at the base of the scapus, taking care not to pull them. Bees with leaking hemolymph were eliminated from the analysis (De Brito Sanchez et al., 2008).

Each subject was checked for intact PER before starting the experiments. This was done by lightly touching the fore tarsi with a toothpick soaked with sucrose solution 1M without subsequent feeding. Care was taken to ensure that the toothpick contacted both the tarsus and the claws (Figure 1). Extension of the proboscis beyond a virtual line between the open mandibles was counted as PER. Animals that did not show the reflex were discarded. Gustatory stimuli were delivered by means of a toothpick soaked in the solution tested. A different toothpick was used for each solution tested.

#### Stimuli

The gustatory stimuli employed in behavioral experiments were distilled water, sucrose 1M, quinine hydrochloride (1/10/100 mM), salicin (1/10/100 mM) and caffeine (1/10/100 mM). All chemicals were obtained from Sigma-Aldrich (Saint-Quentin Fallavier, France).

#### Experiment 1

**Figure 2:**
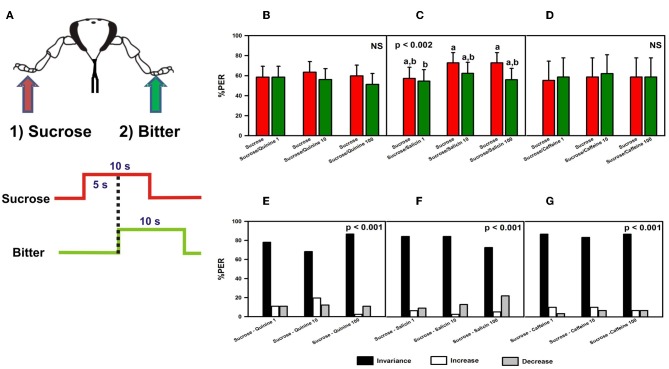
**Experiment 1. (A)** Bees with amputated antennae were stimulated along three trials with sucrose solution 1M (red arrow; red trace) on one fore-tarsus (either left or right) in order to elicit PER, and 5 s later with quinine, salicin, or caffeine (“bitter”, green arrow, green trace) of different concentrations on the contralateral tarsus to determine whether these substances induce proboscis retraction. **(B)** Percentage of proboscis extension responses (% PER) upon stimulation with sucrose 1M (red bars) and quinine (1, 10, and 100 mM; green bars). **(C)** % PER upon stimulation with sucrose 1M (red bars) and salicin (1, 10, and 100 mM; green bars). Different letters above bars indicate significant differences (*p* < 0.05). **(D)** % PER upon stimulation with sucrose 1M (red bars) and caffeine (1, 10, and 100 mM; green bars). **(E)** Percentage of bees into the invariance (black bars), increase (white bars), or decrease (gray bars) categories upon stimulation with sucrose 1M and quinine 1, 10, and 100 mM. “Decrease” means that sucrose induced PER and quinine induced retraction, “increase” means that sucrose did not induce PER but quinine did. “Invariance” means that no response change was induced by quinine with respect to sucrose. **(F)** Percentage of bees into the invariance, increase, or decrease categories upon stimulation with sucrose 1M and salicin 1, 10, and 100 mM. **(G)** Percentage of bees into the invariance, increase, or decrease categories upon stimulation with sucrose 1M and caffeine 1, 10, and 100 mM.

We determined whether bitter substances (quinine, salicin, or caffeine) applied on the fore-tarsi exert an inhibitory effect on proboscis extension reflex (PER), an appetitive response triggered by prior tarsal stimulation with sucrose solution 1 M (Figure 2A). (Kuwabara, 1957; De Brito Sanchez et al., 2005). This sucrose concentration elicits consistent PER when applied to the bees' fore-tarsi (De Brito Sanchez et al., 2008). Bees with amputated antennae were stimulated along three trials with sucrose solution 1 M on one fore-tarsus (either left or right) in order to elicit PER, and with quinine, salicin, or caffeine of different concentrations on the contralateral tarsus to determine whether these substances induce *proboscis retraction* due to their potential aversive nature (Dethier and Bowdan, 1992). Both the first stimulation with sucrose solution and the second stimulation with the bitter substance lasted 10 s. The interstimulus interval was 5 s (onset-onset) (Figure 2A).

We measured the proboscis response during the first 5 s of sucrose stimulation of one fore-tarsus and during the consecutive 5 s in which the second stimulation was delivered to the opposite fore-tarsus (bitter substance). The latter period allowed determining whether or not bees retracted the proboscis. Measuring retraction during the last 5 s of bitter stimulation, in the absence of sucrose, would be inappropriate as retraction could occur simply due to the absence of sucrose and not as a consequence of the bitter stimulation itself.

Three concentrations of bitter substance were consecutively assayed along trials: 1, 10, and 100 mM. The latter corresponds to a highly saturated solution. This increasing sequence was chosen to avoid fast response saturation. To control for possible sensitization induced by sucrose solution, we performed three control trials interspersed between the three bitter-substance trials. In these control trials, bees were stimulated during 10 s with water on one fore-tarsus, and with a dry toothpick during 10 s on the contralateral fore-tarsus. The interstimulus interval was also 5 s.

For each bitter substance, two groups of bees were assayed: for one group, sucrose solution 1 M or water was delivered on the right fore-tarsus and the bitter substance (quinine, salicin, or caffeine) or the dry toothpick on the left fore-tarsus; for the other group, stimulation sites were inversed. Experiments started with a control trial. Control and bitter-substance trials alternated so that each bee was subjected to six trials. The intertrial interval was 10 min.

#### Experiment 2

**Figure 3:**
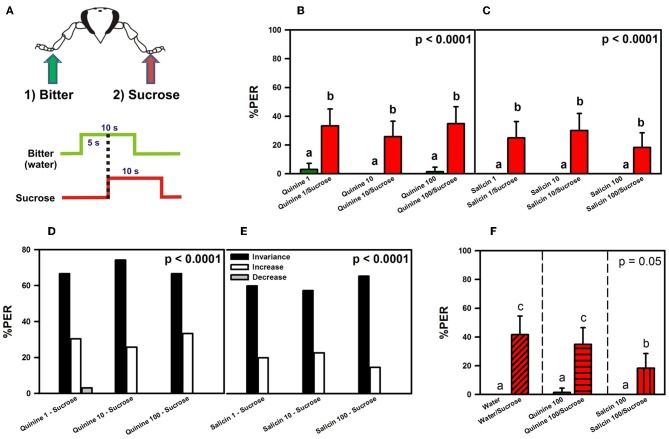
**Experiment 2. (A)** Bees with amputated antennae were stimulated along three trials with quinine or salicin (“bitter”, green arrow, green trace) of different concentrations on one fore-tarsus, and with sucrose solution 1M (red arrow; red trace) on the contralateral fore-tarsus to determine whether sucrose overcomes the potential inhibitory effect of the bitter substance and elicits PER. **(B)** % PER upon stimulation with quinine (1, 10, and 100 mM; green bars) and sucrose 1M (red bars). **(C)** % PER upon stimulation with salicin (1, 10, and 100 mM; green bars) and sucrose 1M (red bars). Different letters above bars indicate significant differences (*p* < 0.05). **(D)** % of bees into the invariance (black bars), increase (white bars), or decrease (gray bars) categories upon stimulation with sucrose 1M and quinine 1, 10, and 100 mM. **(E)** % of bees into the invariance (black bars), increase (white bars), or decrease (gray bars) categories upon stimulation with sucrose 1M and salicin 1, 10, and 100 mM. **(F)** % PER upon stimulation with water and sucrose 1M (diagonally hatched bar), quinine 100 mM and sucrose 1M (horizontally hatched bar) and salicin 100 mM and sucrose 1M (vertically hatched bar). Different letters above bars indicate significant differences (*p* < 0.05).

We studied whether potential PER inhibition by a bitter substance (quinine or salicin) applied on one fore-tarsus can be overcome by sucrose solution 1 M applied on the contralateral fore-tarsus (Figure 3A), thus resulting in PER (Dethier and Bowdan, 1992; Meunier et al., 2003). As in Experiment 1, we used bees with amputated antennae in order to avoid antennal interference (see above). An additional control was performed in which bees with intact antennae were used.

A 10 s stimulation with quinine or salicin solution was applied on one fore-tarsus, followed by a 10 s stimulation with sucrose on the contralateral fore-tarsus. The interstimulus interval was 5 s (onset-onset) (Figure 3A). Proboscis extension was quantified during the 5 s overlap of bitter and sucrose stimulation. Measuring it during the last 5 s of sucrose stimulation, in the absence of bitter stimulation, would be inappropriate as PER would occur simply in response to sucrose alone without overcoming any potential inhibition by the bitter substance.

Three concentrations of bitter substance were consecutively assayed along trials: 1, 10, and 100 mM. The latter corresponds to a highly saturated solution. This increasing sequence was chosen to avoid fast response saturation. To control for possible sensitization effects of sucrose solution, we performed three control trials that were interspersed between the three bitter-substance trials. In these control trials, bees were stimulated during 10 s with water on one fore-tarsus, and with a dry toothpick during 10 s on the contralateral fore-tarsus. The interstimulus interval was also 5 s. Experiments started always with a control trial. Control and bitter-substance trials alternated so that each bee was subjected to six trials. The intertrial interval was 10 min.

Two groups of bees (both for antennae-amputated and intact bees) were treated in this way: for one group, the bitter substance (quinine or salicin) of a given concentration or water was delivered on the right fore-tarsus, and sucrose solution 1 M or the dry toothpick on the left fore-tarsus; for the other group, stimulation sites were inversed.

### Electrophysiological experiments

#### Insects

Captured bees were placed in glass vials and cooled down on ice until they stopped moving. They were then mounted individually in Eppendorf tubes (Le Pecq, France) presenting a lateral slid through which a foreleg could be passed. The fixed bee was laid down horizontally, with the leg extended on a lateral support. The leg was fixed to the support by means of adhesive band tape in order to avoid movements. Bees fixed in this way were kept resting during 1 h before the start of the electrophysiological recordings.

#### Recording sites

The forelegs consist of six segments: the coxa, the trochanter, the femur, the tibia, the tarsus and the pretarsus. Figure 1B shows a detail of these two last segments. The tarsus has five tarsomeres: a basitarsus, which is the largest tarsomere, and 4 smaller tarsomeres (2nd–5^th^). The distally situated pretarsus bears a pair of lateral bifid claws and an arolium, a small pad used to increase adhesion (Figures 1B,C) (Snodgrass, 1956; Goodman, 2003). Approximately 100 taste sensillae are located on the tarsus and pretarsus. These are mostly chaetic sensilla, which are evenly distributed between the five subsegments of the tarsus, and which are densely concentrated on the terminal claw-bearing pretarsus. Chaetic sensilla share similarities with those found on the mouth parts, with a mechanosensory cell ending at their base and four cells with dendrites running to the tip of the shaft (Whitehead and Larsen, 1976b).

Electrophysiological recordings were performed on chaetic sensillae, which could be easily identified by their external morphology and which were located on the third and fourth tarsomeres of the tarsus, and at the level of the claws of the pretarsus.

#### Stimuli

The tastants employed were KCl, NaCl, sucrose, quinine hydrochloride (henceforth quinine), salicin and amygdalin. All chemicals were obtained from Sigma-Aldrich (Saint-Quentin Fallavier, France). Depending on the experiment, chemicals were diluted in a solution of KCl 0.1 or 0.01 mM, which was used as contact electrolyte. Solutions were kept at −4°C. To evaluate the effect of bitter compounds on sensillae responding to sucrose (De Brito Sanchez et al., 2005; Cocco and Glendinning, 2012; Kessler et al., 2012), we tested mixtures of 1M sucrose and either quinine 10 mM or amygdalin 10mM.

#### Single sensillum recordings

A glass electrode with an external diameter of 10–20 μm was placed over a single taste sensillum. Electrodes were pulled from borosilicate glass capillaries. A chlorinated silver wire inserted into the contralateral eye was used as grounded reference electrode. The stimulating electrode was filled with the solution to be assayed (see above). The stimulation electrodes were stored in a humid chamber before use.

Stimuli were applied for 2 s with an interstimulus interval of 1 min. In some experiments stimulation lasted 5 s in order to favor recording of cellular responses to bitter substances such as quinine, which in some insects (e.g., *Heliothis virescens*) exhibit a long latency (Jørgensen et al., 2007). The recording and reference electrodes were connected to a preamplifier (Taste Probe—SYNTECH, Kirchzarten, Germany). The electric signals were amplified (× 10) using a signal connection interface box (SYNTECH, Kirchzarten, Germany) in conjunction with a 100–3000 Hz bandpass filter. Experiments started when the recording electrode contacted the sensillum under study, which triggered data acquisition and storage on a hard disk (sampling rate 10 kHz). These data were then analyzed using Spike 2 and quantified by counting the number of spikes after stimulus onset.

## Results

### Behavioral experiments

#### Experiment 1: induction of proboscis retraction by bitter substances

We determined whether bitter substances applied on a fore-tarsus induce retraction of the proboscis once a proboscis extension reflex (PER) had occurred due to a prior stimulation with sucrose on the other fore-tarsus (Figure 2A). Quinine (*n* = 82), salicin (*n* = 77) and caffeine (*n* = 30) were used as potentially inhibitory substances. There were no significant differences between groups depending on the side (left or right fore-tarsus) of substance stimulation [ANOVA for repeated measurements; quinine: *F*_(1, 80)_ = 0.23, *p* = 0.64; salicin: *F*_(1, 75)_ = 0.11, *p* = 0.75; caffeine: *F*_(1, 28)_ = 1.75, *p* = 0.20] so that data were pooled within each treatment.

Figures 2B–D shows the % of PER during the 5s of sucrose stimulation as reference (red bars), and during the 5s overlap between sucrose and the bitter substance tested (green bars). In the case of quinine (Figure 2B), no retraction of proboscis was observed upon stimulation with all three concentrations tested. Response remained unchanged with respect to that elicited by sucrose solution [*F*_(5, 405)_ = 0.83, *p* = 0.53], thus showing that quinine did not exert an inhibitory effect on a prior response to sucrose solution. Responses to control stimulations with water and dry toothpick (not shown) remained constant and low along trials [*F*_(5, 405)_ = 1.06, *p* = 0.38] and were significantly lower than those to sucrose and quinine [*F*_(1, 81)_ = 163.28, *p* < 0.0001]. In the case of salicin, despite significant differences between trials [*F*_(5, 380)_ = 3.88, *p* < 0.002], responses to sucrose never decreased significantly within a trial due to salicin stimulation (Figure 2C). Responses to control stimulations with water and the dry toothpick (not shown) were homogeneous [*F*_(5, 380)_ = 1.65, *p* = 0.15] and significantly lower than those to sucrose and salicin [*F*_(1, 76)_ = 203.20, *p* < 0.0001]. Finally, caffeine was also ineffective to induce proboscis retraction, irrespective of the concentration used [Figure 2D: *F*_(5, 140)_ = 0.10, *p* = 0.99]. In this case, control stimulations with water and the dry toothpick induced heterogeneous responses [*F*_(5, 140)_ = 3.84, *p* < 0.003; not shown] but these were significantly lower than those induced by sucrose and caffeine [*F*_(1, 28)_ = 54.19, *p* < 0.0001]. In all cases, the level of PER to sucrose was high, thus revealing a high appetitive motivation.

In order to refine the analyses on the basis of individual responses, we distinguished three main classes of responses occurring within a trial: “*decrease*” (1 → 0), in which sucrose induced PER and the bitter substance induced retraction, “*increase*” (0 → 1), in which sucrose did not induce PER but the bitter substance did, and “*invariance*”, in which no response change was induced by the bitter substance with respect to sucrose solution (0 → 0 and 1 → 1). Upon stimulation with sucrose and quinine (Figure 2E), most of the bees (78% in average) fell into the *invariance* category (black bars), thus confirming that irrespective of the concentration of quinine used these bees did not change their response upon tarsal quinine contact. Only 11% of bees in average fell into the *increase* (white bars) or the *decrease* category (gray bars). Differences between categories were significant within each trial (χ^2^ test, *p* < 0.01 in all cases). A similar conclusion was reached in the case of bees stimulated with sucrose and salicin (Figure 2F). Eighty-one percent of the bees fell into the invariance category, 4% into the increase category and 14% into the decrease category. Differences between categories were significant within each trial (χ^2^ test, *p* < 0.01 in all cases). In the case of stimulation with sucrose and caffeine (Figure 2G), 86% of the bees fell into the *invariance* category, 9% into the *increase* category and 6% into the *decrease* category. Differences between categories were significant within each trial (χ^2^ test, *p* < 0.01 in all cases). Thus, for all three substances, the low percentages corresponding to the *decrease* category showed that the bitter substances tested did not induce significant proboscis retraction after sucrose stimulation.

#### Experiment 2: inhibition of appetitive proboscis extension by bitter substances

We analyzed whether stimulation with sucrose solution on a fore-tarsus overcomes a potential inhibitory effect of stimulation with a bitter-substance on the other fore-tarsus and thus triggers PER. Quinine (*n* = 66) and salicin (*n* = 60) were used as potentially inhibitory substances. There were no significant differences between groups depending on the side (left or right fore-tarsus) of substance stimulation [ANOVA for repeated measurements; quinine: *F*_(1, 64)_ = 0.11, *p* = 0.74; salicin: *F*_(1, 58)_ = 0.37, *p* = 0.54] so that data were pooled within each treatment.

Figures 3B,C shows the % of PER during the 5s of bitter stimulation as reference (green bars), and during the 5s overlap between sucrose and the bitter substance tested (red bars). Figure 3B shows that bees did not extend the proboscis upon quinine contact with one fore tarsus, irrespective of the concentration used. Simultaneous stimulation with quinine and sucrose solution on opposite fore tarsi induced a significant level of PER thus showing that quinine did not inhibit PER [*F*_(5, 325)_ = 18.73, *p* < 0.00001]. Responses to quinine were all equivalent (Tukey *post hoc* tests; NS for all comparisons) and significantly different from those to sucrose solution (Tukey *post hoc* tests; *p* < 0.001 for all comparisons), which were also equivalent between trials (Tukey *post hoc* tests; NS for all comparisons). Responses to control stimulations with water and the dry toothpick (not shown) remained constant and low along trials [*F*_(5, 325)_ = 1.68, *p* = 0.14]. Figure 3C shows a similar pattern of responses upon stimulation with salicin and sucrose: bees did not extend the proboscis upon salicin contact with one fore tarsus, irrespective of the concentration of salicin solution used, while they extended the proboscis upon simultaneous stimulation with sucrose solution on the opposite fore tarsus [*F*_(5, 295)_ = 13.03, *p* < 0.00001]. Responses to salicin were all equivalent (Tukey *post hoc* tests; NS for all comparisons) and significantly different from those to sucrose solution (Tukey *post hoc* tests; *p* < 0.001 for all comparisons), which were also equivalent between trials (Tukey *post hoc* tests; NS for all comparisons). Responses to control stimulations (not shown) remained constant and low along trials [*F*_(5, 925)_ = 1.68, *p* = 0.14].

An analysis of individual responses in terms of the three classes of response variations, “*decrease*” (1 → 0), “*increase*” (0 → 1) and “*invariance*” (0 → 0 and 1 → 1), showed that upon stimulation with quinine and sucrose (Figure 3D), 69% of the bees in average fell into the *invariance* category, 30% into the *increase* category and 1% into the *decrease* category. Differences between the three categories were significant within each trial (χ^2^ test, *p* < 0.01 in all cases). Stimulation with salicin resulted in 76% of bees in average in the *invariance* category and 24% in the *increase* category. No bee fell into the *decrease* category (Figure 3E). Differences between the three categories were significant within each trial (χ^2^ test, *p* < 0.01 in all cases).

**Figure 4:**
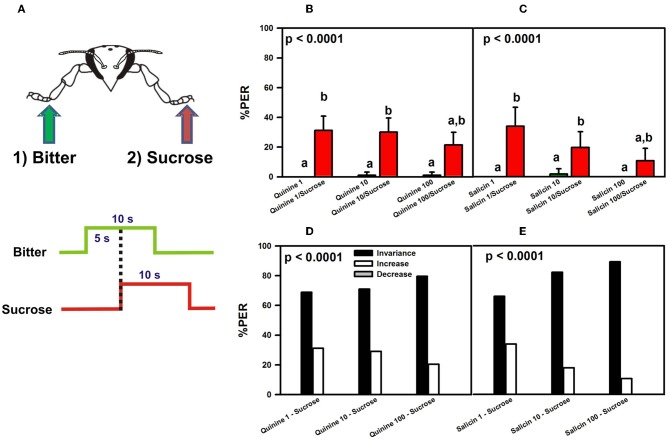
**Experiment 2. (A)** Bees with intact antennae were stimulated along three trials with quinine or salicin (“bitter,” green arrow, green trace) of different concentrations on one fore-tarsus, and with sucrose solution 1M (red arrow; red trace) on the contralateral fore-tarsus to determine whether sucrose overcomes the potential inhibitory effect of the bitter substance and elicits PER. **(B)** % PER upon stimulation with quinine (1, 10, and 100 mM; green bars) and sucrose 1M (red bars). **(C)** % PER upon stimulation with salicin (1, 10, and 100 mM; green bars) and sucrose 1M (red bars). Different letters above bars indicate significant differences (*p* < 0.05). **(D)** % of bees into the invariance (black bars), increase (white bars), or decrease (gray bars) categories upon stimulation with sucrose 1M and quinine 1, 10, and 100 mM. **(E)** % of bees into the invariance (black bars), increase (white bars), or decrease (gray bars) categories upon stimulation with sucrose 1M and salicin 1, 10, and 100 mM.

These results might suggest that quinine and salicin are aversive and inhibit PER elicited by concomitant stimulation with sucrose because the average % of PER recorded was low (30% upon quinine and sucrose stimulation, and 24% upon salicin and sucrose stimulation). Yet, the low percentages of responsiveness to sucrose could be due to antennal amputation rather than reflecting an aversive nature of quinine and salicin. Antennal amputation has been shown to decrease sucrose responsiveness upon tarsal stimulation (De Brito Sanchez et al., 2008) so that the previous experiment was repeated with intact bees (Figure 4A).

Quinine (*n* = 93) and salicin (*n* = 56) were again used as potentially inhibitory substances. There were no significant differences between groups depending on the side (left or right fore-tarsus) of substance stimulation [ANOVA for repeated measurements; quinine: *F*_(1, 91)_ = 1.53, *p* = 0.22; salicin: *F*_(1, 54)_ = 0.43, *p* = 0.51] so that data were pooled within each treatment.

The pattern of responses of intact bees upon stimulation with quinine and sucrose (Figure 4B) was similar to that exhibited by bees which had their antennae cut (Figure 3B). Bees did not extend their proboscis upon quinine contact with one fore tarsus, irrespective of the concentration of quinine solution used, while they extended their proboscis upon simultaneous stimulation with quinine and sucrose solution on opposite fore tarsi [*F*_(5, 460)_ = 23.12, *p* < 0.00001]. Responses to quinine were all equivalent (Tukey *post hoc* tests; NS for all comparisons) and significantly different from those to sucrose solution (Tukey *post hoc* tests; *p* < 0.001 for all comparisons), which were also equivalent between trials (Tukey *post hoc* tests; NS for all comparisons). Similarly to the experiment with amputated bees, the average % of PER to sucrose upon simultaneous stimulation with quinine and sucrose solution was 28%.

Similarly to antenna-amputated bees (Figure 3C), intact bees did not extend their proboscis upon contact of one fore tarsus with salicin solution (Figure 4C), irrespective of the concentration used, while they extended their proboscis upon simultaneous stimulation with salicin and sucrose solution on opposite fore tarsi [*F*_(5, 275)_ = 12.62, *p* < 0.00001]. As in the experiment with antenna-amputated bees, the average % of PER to sucrose upon simultaneous stimulation with salicin and sucrose solution was 21%.

The analysis of individual responses of intact bees in terms of the *increase*, *decrease*, and *invariance* categories yielded the same results as those obtained in antenna-amputated bees stimulated with quinine (Figure 4D) or salicin (Figure 4E). Stimulation with quinine resulted in 73% of bees in average in the *invariance* category, 27% in the *increase* category and 0% in the *decrease* category. Stimulation with salicin determined that 79% of the bees fell into the *invariance* category, 21% into the *increase* category and 0% into the *decrease* category.

Thus, the low percentages of responsiveness to sucrose were not due to antennal amputation as the same percentages were obtained in experiments in which bees conserved their antennae. To determine whether the two bitter substances did indeed exert a partial inhibitory effect on PER upon sucrose stimulation due to their aversive nature, we performed a final control experiment with antenna-amputated bees in which we replaced the first stimulation with a bitter substance by stimulation with water on one fore tarsus, followed by stimulation with sucrose solution on the opposite fore tarsus (Figure 3F).

No significant differences were found between the subgroup that received water on the left fore tarsus (and sucrose on the right fore tarsus) and that receiving water on the right fore tarsus (and sucrose on the left fore tarsus) [*F*_(1, 58)_ = 0.55, *p* = 0.46] so that results of both subgroups were pooled (Figure 3F). The responses of two other groups run in parallel and stimulated with quinine 100 mM and sucrose, and with salicin 100 mM and sucrose, are also shown.

Bees did not show PER to water alone upon contact with one fore-tarsus, while they showed it when sucrose contacted the other fore tarsus. The % of PER upon simultaneous stimulation with water and sucrose on opposite fore tarsi was, however, only 40% so that responses of the water group did not differ significantly from those of the quinine and the salicin groups even if the lack of significance was marginal [*F*_(2, 183)_ = 3.01, *p* = 0.05]. *Post hoc* analyses showed that responses to water, quinine and salicin alone did not differ significantly (Tukey tests; all comparisons NS) while responses to simultaneous stimulation with water and sucrose, and quinine and sucrose were significantly higher than responses to water, quinine and salicin alone (Tukey tests; all comparisons *p* < 0.05). Responses to simultaneous stimulation with salicin and sucrose reached an intermediate level. Thus, simultaneous stimulation with water and sucrose induced the same effect on PER as simultaneous stimulation with quinine and sucrose. The analysis of individual responses (not shown) yielded 60% of bees in the invariance category, 40% in the increase category and 0% in the decrease category.

The lower levels of PER registered in all experiments (Figures 3 and 4) seem, therefore, due to contradictory gustatory input from both fore tarsi and not to a potential aversive nature of bitter substances. The fact that water yielded the same pattern of responses as concentrated quinine solution confirms that sensitivity for bitter substances is rather limited at the tarsal level. Only the fact that concentrated salicin solution induced a lower level of PER upon sucrose stimulation compared to quinine solution and water (Figure 3F) indicates some inhibitory effect of salicin.

### Electrophysiological experiments

#### Experiment 3: responses of tarsomere sensilla to sweet, bitter and salty substances

**Figure 5:**
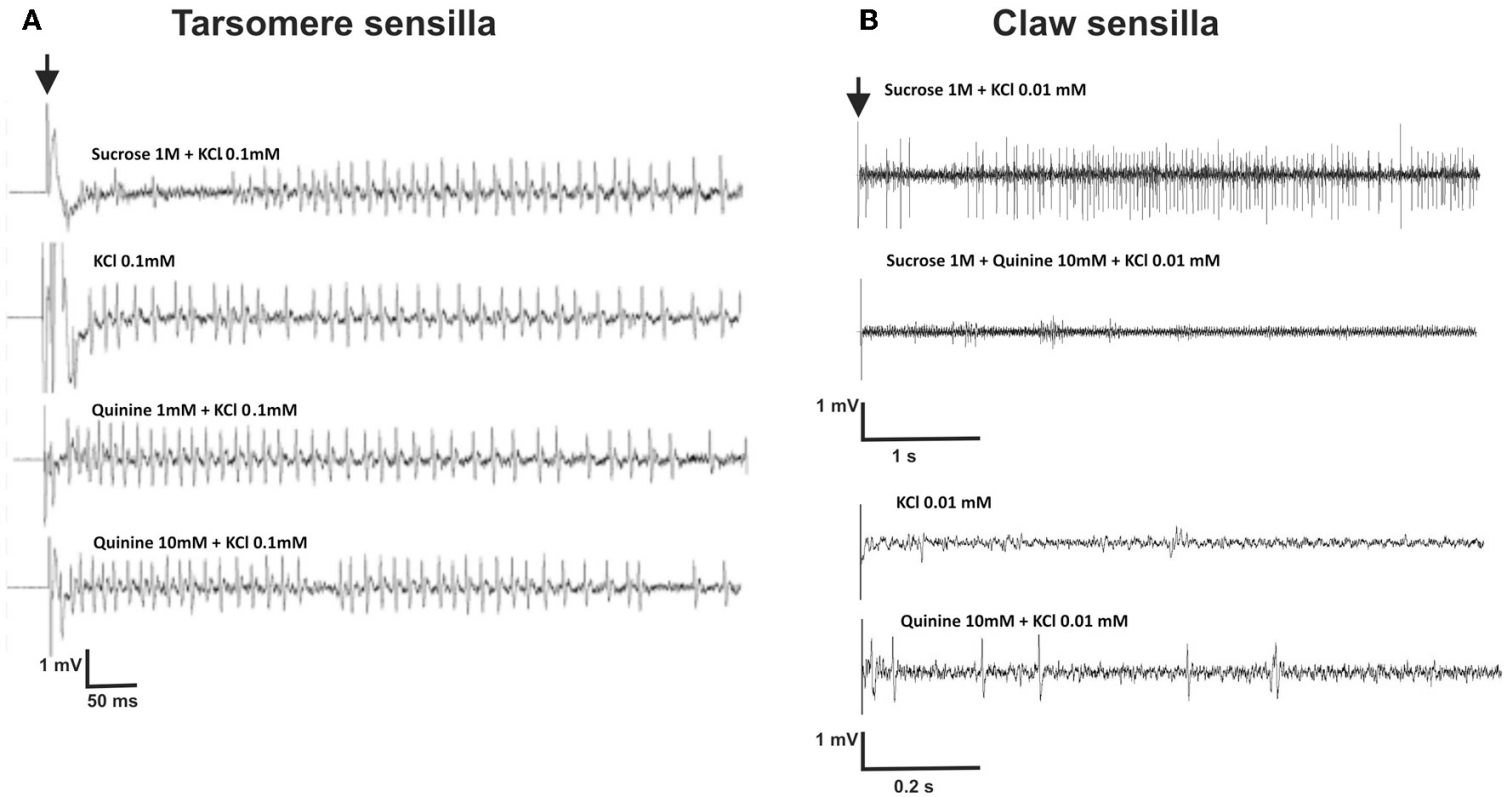
**(A)** Experiment 3: Examples of extracellular recordings performed at the level of chaetic sensilla located on the third and fourth tarsomeres of the foreleg, upon 2-s gustatory stimulation. The contact electrolyte used for all solutions was KCl 0.1 mM. The black arrow indicates the start of stimulation. Vertical scale: 1 mV; horizontal scale: 50 ms. **(B)** Experiment 6: Examples of extracellular recordings performed at the level of chaetic sensilla located on the claws, upon 5-s gustatory stimulation.

We recorded responses of gustatory receptor neurons located in chaetic sensilla of the third and fourth tarsomeres of the foreleg, upon 2-s stimulation with sucrose 1 M, quinine solution 1 and 10 mM, salicin 1 mM, KCl 0.1 mM and NaCl 100 mM. The contact electrolyte used for all solutions was KCl 0.1 mM which proved to be effective and did not elicit significant spiking activity *per se* in recordings of chaetic sensilla performed at the level of the antenna (De Brito Sanchez et al., 2005). Left and right fore legs were used indistinctly. Figure 5A shows examples of recordings obtained. Contrary to our expectations, the contact electrolyte (0.1 mM KCl) elicited significant spiking activity (Figure 5A) even if higher concentrations of KCl (e.g., 10 mM) have been used as contact electrolyte to study gustatory responses in other appendages such as the antennae of bees and moths without inducing significant neural activity (De Brito Sanchez et al., 2005; Jørgensen et al., 2007). As shown by Figure 5A, recordings obtained upon stimulation with different tastants such as KCl and quinine showed a striking similarity and rendered difficult distinguishing between different receptor neurons based on the amplitude of action potentials.

**Figure 6:**
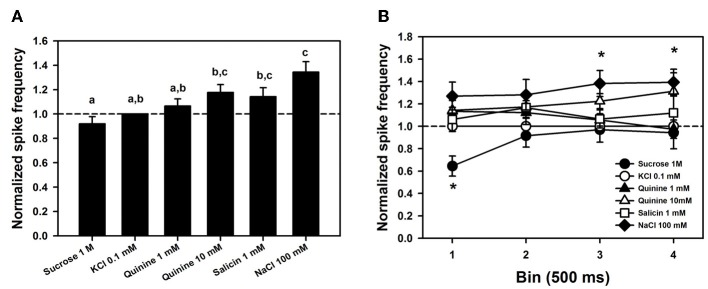
**(A)** Mean normalized spike frequencies (±SE) obtained upon stimulation with the different tastants assayed. Responses (action potentials per second) were normalized to those recorded for KCl 0.1 mM (hatched horizontal line). Data were obtained from 6 bees in which responses for the six tastants assayed were recorded 4 times (*n* = 144) in 7 different sensilla. Different letters above bars indicate significant differences (*p* < 0.05). **(B)** Temporal analysis of mean normalized spike frequencies along four consecutive bins of 500 ms each, starting at the onset and finishing at the offset of stimulation. Asterisks indicate significant differences between tastants within a bin (*p* < 0.05).

Figure 6A shows mean responses (±SE) to the different tastants, normalized to the response obtained for the contact electrolyte alone (KCl 0.1 mM). This normalization is necessary because levels of response may differ considerably between individuals. Data were obtained from 6 bees in which responses for the six tastants assayed were recorded 4 times (*n* = 144) in 7 different sensilla. Normalized responses varied significantly depending on the tastant assayed [Figure 6A; ANOVA for repeated measurements: *F*_(5, 115)_ = 9.02, *p* < 0.0001]. *Post hoc* analyses (Tukey test) showed that responses to sucrose solution 1 M were significantly lower than those to NaCl 100 mM, quinine 10 mM and salicin 1mM (Tukey tests: *p* < 0.05 for all three comparisons). Responses to the three bitter solutions (quinine 1 and 10 mM and salicin 1mM) did not differ (Tukey test: *p* > 0.05 for all three comparisons) and were also indistinguishable from responses to the contact electrolyte KCl 0.1 mM that was also present in these bitter solutions (*p* > 0.05 for all three comparisons). These results suggest, therefore, both a lower sensitivity for sucrose and a higher sensitivity for NaCl and that responses to quinine and salicin could be, in fact, responses to the KCl present in these solutions.

To determine whether responses to tastants could be distinguished from those to KCl in terms of their temporal course, we analyzed normalized responses along four consecutive bins of 500 ms each, starting at the onset and finishing at the offset of stimulation (Figure 6B). No significant differences between bins were detected [*F*_(3, 18)_ = 0.60, *p* = 0.63] but differences between tastants existed [*F*_(5, 30)_ = 6.82, *p* < 0.0005]. These differences were due to responses to sucrose, which were significantly lower than responses to the three bitter substances and NaCl in the 1st bin (*p* < 0.005 for all four comparisons). No significant differences were found within the 2nd bin, while in the 3rd and 4th bins, responses to NaCl 100 mM were significantly higher than those to sucrose 1 M and than those to sucrose 1 M, KCl 0.1 mM and quinine 1 mM, respectively. Responses to NaCl 100 mM remained high along bins, and except for the 2nd bin, were significantly higher than those to sucrose 1 M. No significant differences between responses to KCl and to bitter substances were found along the four bins (*p* > 0.05 for all comparisons), thus showing that temporal dynamics does not allow concluding on the existence of tarsomere cells tuned to bitter substances. Neither the form of action potentials, nor their size or frequency allowed separating responses to KCl from responses to the bitter tastants quinine and salicin. A similar conclusion may apply to sucrose detection: except for the first bin, spike activity upon sucrose stimulation did not differ from that elicited by KCl 0.1 mM, thus suggesting that the low activity recorded upon sucrose stimulation was due to the contact electrolyte.

#### Experiment 4: dose-response curve of tarsomere sensilla for KCl

The previous results suggest that responses to sucrose and to bitter substances of gustatory receptor neurons located in chaetic sensilla of the third and fourth tarsomeres of the foreleg were due to a receptor cell responding to KCl at very low concentrations.

**Figure 7:**
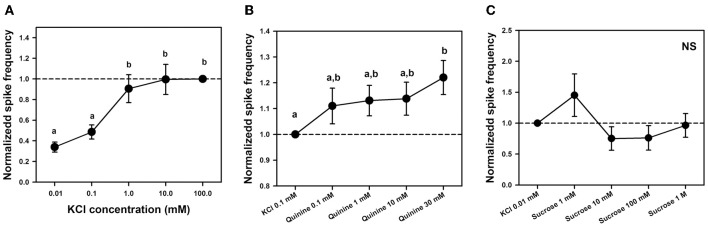
**(A)** Experiment 4. Mean normalized spike frequencies (±SE) obtained upon stimulation with 5 different concentrations of KCl solution: 0.01, 0.1, 1, 10, and 100 mM. Responses (action potentials per second) were normalized to those recorded for KCl 100 mM (hatched horizontal line), which yielded maximal responsiveness. Data were obtained from 6 bees in which responses to the 5 KCl concentrations were recorded 4 times in 6 different sensilla (*n* = 120). Different letters above bars indicate significant differences (*p* < 0.05). **(B)** Experiment 5. Mean normalized spike frequencies (±SE) obtained upon stimulation with KCl 0.1 mM and 4 different concentrations of quinine solution including KCl 0.1 mM as contact electrolyte: 0.1, 1, 10, and 30 mM. Responses (action potentials per second) were normalized to those recorded for KCl 0.1 mM (hatched horizontal line). Data were obtained from 8 bees in which responses to the 5 substances assayed were recorded 4 times in 9 different sensilla (*n* = 180). Different letters above bars indicate significant differences (*p* < 0.05). **(C)** Experiment 5: Mean normalized spike frequencies (±SE) obtained upon stimulation with KCl 0.01 mM and 4 different concentrations of sucrose solution—1 mM, 10 mM, 100 mM and 1 M—including KCl 0.01 mM as contact electrolyte. Responses (action potentials per second) were normalized to those recorded for KCl 0.01 mM (hatched horizontal line). Stimulations lasted 1 s. The graph shows the responses of 14 different sensilla of 5 bees to the 5 substances assayed, each one being tested once (*n* = 70). No differences between stimuli were found; NS, non-significant.

To determine whether a KCl-receptor cell existed within the sensilla studied, we performed an experiment aimed at establishing a dose-response curve to KCl. We quantified responses to 2-s stimulations with five increasing concentrations of KCl: 0.01, 0.1, 1, 10, and 100 mM. Responses were standardized to responses to KCl 100 mM which induced in all cases maximal responsiveness. Figure 7A shows the responses of 6 different sensilla of 6 bees to the five KCl concentrations, each one being tested 4 times (*n* = 120).

Responses increased significantly with KCl concentration [*F*_(4, 92)_ = 16.10, *p* < 0.0001]; specifically, responses to the two lower KCl concentrations (0.01 and 0.1 mM) were significantly lower than those elicited by the three higher concentrations (1, 10, and 100 mM, *p* < 0.005 for all six comparisons; Figure 7A). This variation, which is incompatible with the response of a water receptor cell, shows that the discharge rate observed for the concentration of 0.1 mM KCl used as contact electrolyte in the previous experiment was due to the existence of at least one KCl cell. It also suggests that responses to both bitter substances and sucrose were in fact due to the presence of this electrolyte in the solutions rather than to these substances themselves. The next experiment analyzed this possibility.

#### Experiment 5: dose-response curve of tarsomere sensilla for quinine and sucrose

To determine whether tarsomere sensilla host specific gustatory receptor neurons tuned to bitter substances and sucrose, we aimed at establishing dose-response curves for quinine, on the one hand, and for sucrose on the other hand.

To establish a dose-response curve for quinine, we varied quinine concentration but kept constant the concentration of the contact electrolyte (KCl 0.1 mM) in order to determine whether responses increased progressively, consistently with the presence of a bitter receptor within the sensilla studied, or remained constant if they were due to the KCl cell. We recorded responses to KCl 0.1 mM and to solutions of quinine 0.1, 1, 10, and 30 mM, all containing KCl 0.1 mM. Stimulations lasted 5 s due to the potential long latency of a putative “quinine cell” (Jørgensen et al., 2007). Preliminary experiments showed that the highest concentration of quinine (30 mM) was at the limit of cell tolerance as it could induce higher discharge rates followed by cell death in some cases. Figure 7B shows the responses (spikes/seconds, normalized to KCl responses) of 9 different sensilla of 8 bees to the 5 substances assayed, each one being tested 4 times (*n* = 180).

Responses normalized to those induced by the contact electrolyte increased significantly with quinine concentration [Figure 7B: *F*_(4, 140)_ = 3.98, *p* < 0.005]. Yet *post hoc* analyses indicated that significance was introduced by the comparison between the highest concentration of quinine assayed (30 mM) and KCl 0.1 mM alone (Tukey test: *p* < 0.001). All other comparisons were non-significant. When the analysis was circumscribed to all four quinine concentrations (0.1–30 mM), no significant differences were detected [*F*_(3, 105)_ = 1.92, *p* = 0.13]. The increase in responses found for quinine 30 mM with respect to KCl alone could be due to enhanced responsiveness following cell damage by excessive quinine concentration. The fact that no significant variation existed between all quinine concentrations over two orders of magnitude shows that responses to quinine solutions were in fact due to the presence of gustatory receptor cells responding to the contact electrolyte KCl 0.1 mM, which was common to the bitter tastants assayed.

To establish a dose-response curve for sucrose, we diminished the concentration of the contact electrolyte to 0.01 mM to avoid interferences from the salt in the sucrose responses and to better visualize these responses. We varied sucrose concentration but kept constant the concentration of KCl in order to determine whether responses increased progressively because of the presence of a sucrose receptor cell, or remained constant because they were due to the KCl cell. We recorded responses to KCl 0.01 mM and to solutions of sucrose 1, 10, 100, and 1 M, all containing KCl 0.01 mM. Stimulations lasted 1 s. Figure 7C shows the responses (spikes/seconds, normalized to KCl responses) of 14 different sensilla of 5 bees to the 5 substances assayed, each one being tested once (*n* = 70).

Responses normalized to those induced by the contact electrolyte did not vary significantly with sucrose concentration over four orders of magnitude (Figure 7C). When the analysis was circumscribed to all four sucrose concentrations (1 mM–1 M), no significant differences were detected [*F*_(3, 39)_ = 2.46, *p* = 0.08] despite the apparent increase of responses to sucrose 1 mM. This result indicates that activity elicited by sucrose solution was in fact due to the presence of gustatory receptor cells responding to the contact electrolyte KCl 0.01 mM.

#### Experiment 6: responses of claw sensilla to sucrose, bitter substances and mixtures of sucrose and bitter substances

We finally aimed at determining the sensitivity of chaetic sensilla located on the claws to perform a comparative analysis with those recorded on the tarsomeres. We used KCl 0.01 mM as contact electrolyte and tested the effect of KCl, sucrose, quinine, amygdalin, and mixtures of sucrose and quinine, and of sucrose and amygdalin. Responses were normalized to response levels obtained for the contact electrolyte.

**Figure 8:**
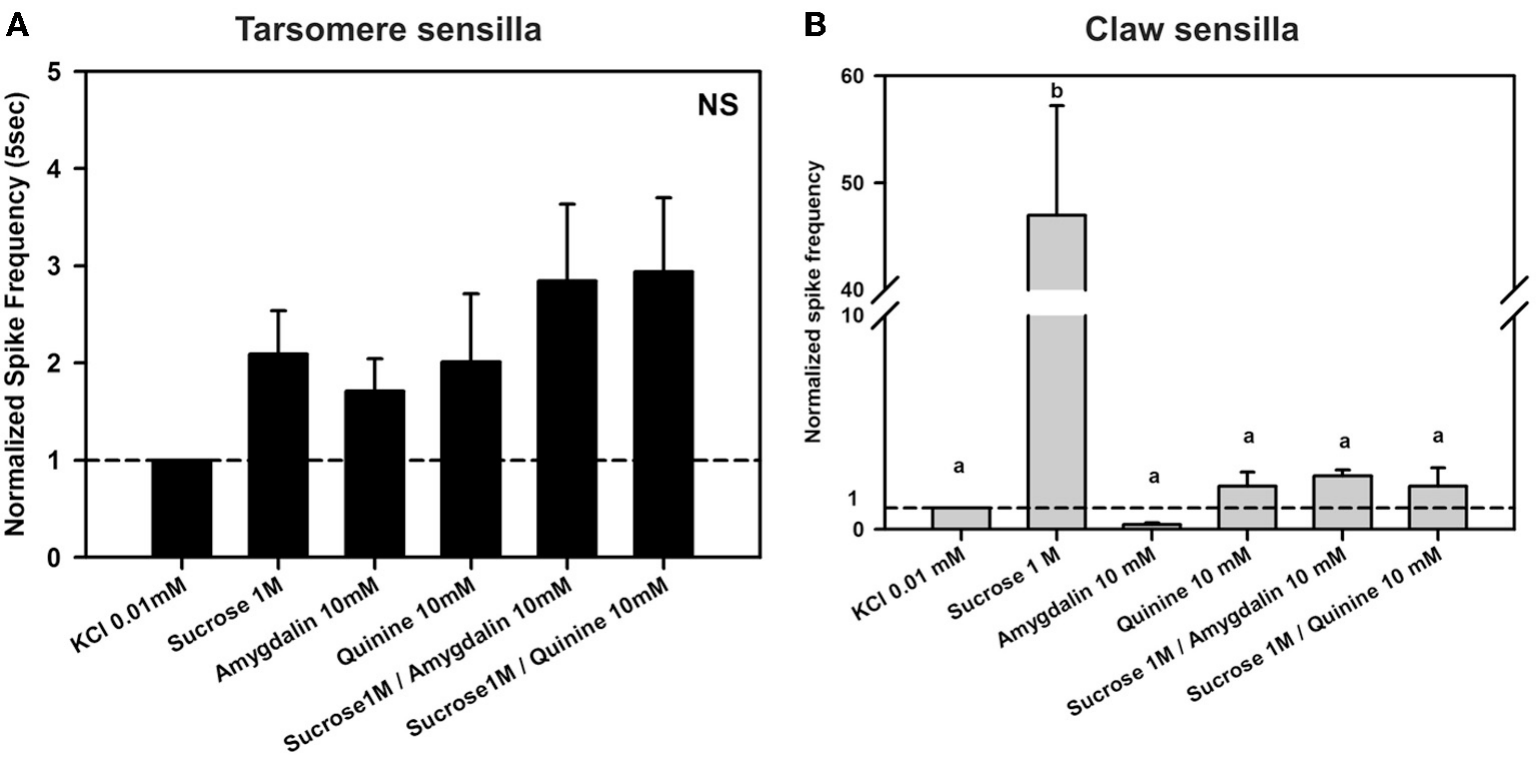
**Experiment 6. (A)** Chaetic sensilla of the 3rd and 4th tarsomeres. Mean normalized spike frequencies (±SE) obtained upon stimulation with the different tastants assayed. Responses (action potentials per second) were normalized to those recorded for KCl 0.01 mM (hatched horizontal line). Data were obtained from 12 tarsomere sensilla of 5 bees in response to the 6 stimuli assayed, each one being tested once (*n* = 72). NS, non significant. **(B)** Chaetic sensilla of the claws. Mean normalized spike frequencies (±SE) obtained upon stimulation with the different tastants assayed. Responses (action potentials per second) were normalized to those recorded for KCl 0.01 mM (hatched horizontal line). Data were obtained from 13 claw sensilla of 4 bees in response to the 6 stimuli assayed, each one being tested once (*n* = 78). Different letters above bars indicate significant differences (*p* < 0.05).

Figure 5B shows examples of recordings obtained for claw sensilla. Contrary to recordings obtained for tarsomere sensilla (Figure 5A), responses to sucrose 1M were clearly different from those obtained for the contact electrolyte KCl 0.01 mM. Figures 8A,B shows mean responses (±SE) of claw sensilla and of tarsomere sensilla to the different tastants, normalized to the response obtained for the contact electrolyte (KCl 0.01 mM). In order to facilitate comparisons between claw and tarsomere sensilla, recordings of tarsomere sensilla were performed again, using KCl 0.01 mM as contact electrolyte (in our previous characterization of these sensilla KCl 0.1 mM was used). For both kinds of sensilla, stimulations lasted 5 s.

Figure 8A shows the normalized spike frequencies of 12 tarsomere sensilla of 5 bees in response to the 6 stimuli assayed, each one being tested once (*n* = 72). Responses to the different stimuli did not vary significantly [*F*_(5, 55)_ = 2.32, *p* = 0.06]. As in Experiment 3, responses to sucrose 1 M were similar to those induced by KCl (*p* = 0.59; see also Figure 6A) even if the contact electrolyte was now diluted in one order of magnitude. Furthermore, responses induced by the bitter substances quinine 10 mM and amygdalin 10 mM did also not differ from those recorded to the electrolyte alone and to sucrose 1M (*p* = 0.90 and 0.67, respectively). Both mixtures of quinine and sucrose and amygdalin and sucrose did not induce any change in responses with respect to the bitter substance or the sucrose alone (*p* > 0.05 for all comparisons).

In the case of claw sensilla, a different pattern of responses was obtained. Figure 8B shows the normalized spike frequencies of 13 claw sensilla of 4 bees in response to the 6 stimuli assayed, each one being tested once (*n* = 78). Normalized spike frequencies varied significantly with the stimulus assayed [*F*_(5, 60)_ = 5.11, *p* < 0.001]. While responses to the bitter substances quinine 10 mM and amygdalin 10 mM did not differ from those to the contact electrolyte KCl 0.01 mM (*p* = 1 for both comparisons), responses to sucrose solution 1M were significantly higher than those induced by these three tastants (*p* < 0.01 for all 3 comparisons), thus suggesting that a sensitive sucrose receptor cell exists within chaetic sensilla of the claws. This receptor was inhibited by quinine 10 mM as shown by the fact that responses to sucrose 1 M and to the mixture of sucrose 1 M and quinine 10 mM differed significantly (*p* < 0.01). In fact, the addition of quinine to the sucrose solution lowered the response to the same level as that of the contact electrolyte KCl 0.01 mM (*p* = 1). The other bitter substance, amygdalin 10 mM, did not induce inhibition of the sucrose receptor; responses to sucrose 1 M and to the mixture of sucrose 1 M and amygdalin 10 mM did not differ significantly (*p* = 0.83).

**Figure 9:**
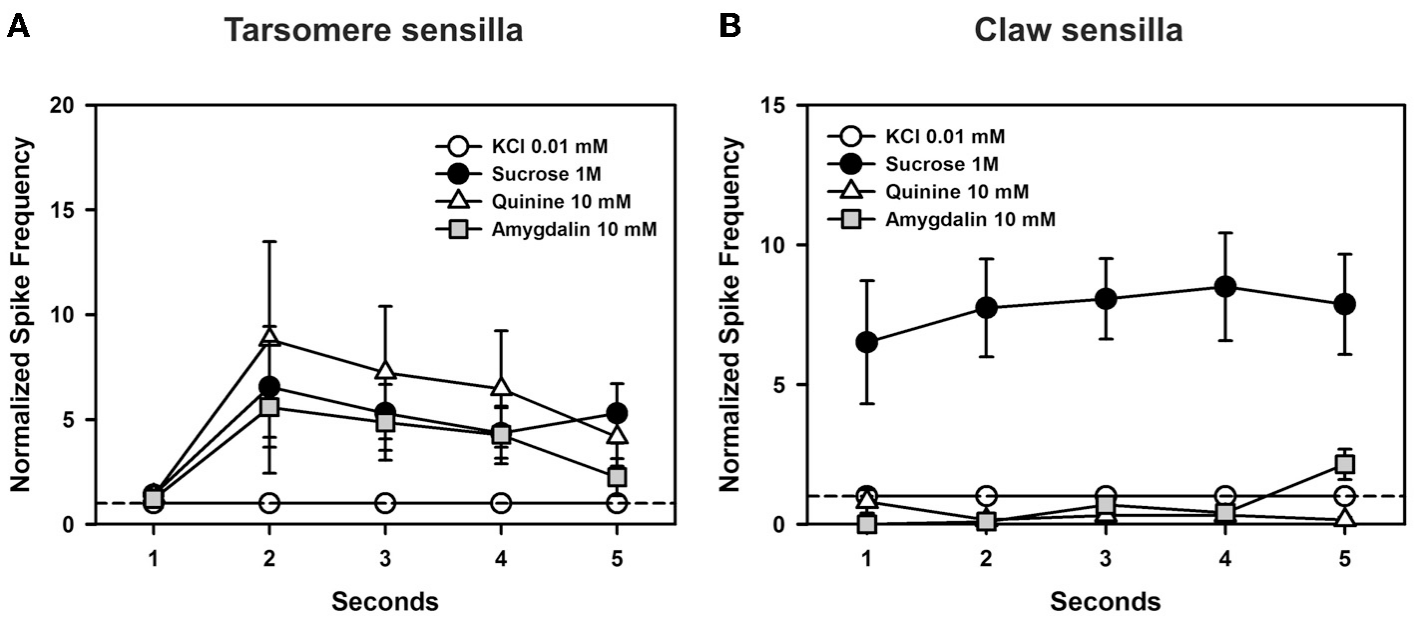
**Experiment 6. (A)** Chaetic sensilla of the 3rd and 4th tarsomeres. Temporal analysis of mean normalized spike frequencies along five consecutive seconds of stimulation, starting at the onset and finishing at the offset of stimulation. The hatched line shows the reference response to the contact electrolyte KCl 0.01 mM. Besides KCl, the curves show the temporal responses upon stimulation with sucrose 1 M, quinine 10 mM, and amygdalin 10 mM. There were significant differences between seconds of stimulation (*p* < 0.0005) but within each second, the responses obtained for the different tastants were statistically similar. **(B)** Chaetic sensilla of the claws. Temporal analysis of mean normalized spike frequencies along five consecutive seconds of stimulation, starting at the onset and finishing at the offset of stimulation. The hatched line shows the reference response to the contact electrolyte KCl 0.01 mM. Besides KCl, the curves show the temporal responses upon stimulation with sucrose 1 M, quinine 10 mM, and amygdalin 10 mM. Responses varied significantly with the substance assayed as responses to sucrose 1 M were higher than those to the other tastants all along the stimulation period (*p* < 0.0001). Differences were neither found for the factor “second” of stimulation nor for the interaction “substance × second”.

The differences between tarsomere and claw sensilla can also be visualized by comparing the temporal course of their responses to the tastants assayed. To this end, we analyzed normalized responses along the 5 s of stimulation (from stimulus onset to offset) (Figure 9). In the case of tarsomere sensilla (Figure 9A), neither the factor “substance” [*F*_(3, 44)_ = 1.88, *p* = 0.15] nor the interaction “substance × second” was significant [*F*_(12, 176)_ = 0.88, *p* = 0.56], thus showing that for all substances assayed the temporal pattern of responses was similar. There were significant differences between seconds of stimulation [factor “second”: *F*_(4, 176)_ = 5.35, *p* < 0.0005] but within each second, the responses obtained for the different tastants were statistically similar. This result confirms the prior temporal analysis performed on tarsomere sensilla (see Figure 6B), which showed that quinine 1 and 10 mM, salicin 1 mM and sucrose 1M yielded comparable pattern of responses as the contact electrolyte KCl 0.1 mM. With a more diluted contact electrolyte (KCl 0.01 mM; Figure 9A), normalized responses were somehow higher (compare Figures 6B and 9A), yet undistinguishable from those obtained for the contact electrolyte. These results thus confirm the absence of bitter-tuned and sucrose-tuned receptor cells at the level of the tarsomere sensilla.

In the case of claw sensilla (Figure 9B), there were significant differences in cell responses to the substances assayed [*F*_(3, 48)_ = 20.93, *p* < 0.0001]. Differences were neither found for the factor “second” of stimulation [*F*_(4, 192)_ = 0.89, *p* = 0.47] nor for the interaction “substance × second” [*F*_(12, 192)_ = 1.05, *p* = 0.43]. These results thus show that during the 5 s of stimulation, responses to sucrose were higher than those to other tastants. Responses to quinine and amygdalin were low and undistinguishable from those elicited by KCl.

## Discussion

The tarsal taste of the honey bee has remained until now mostly unexplored (De Brito Sanchez, 2011) despite its long-claimed importance in taste detection (Frings and Frings, 1949). The present work provides the first integrative account on honey bee tarsal responses to sweet, salty, and bitter substances both at the behavioral and electrophysiological levels.

### Behavioral and electrophysiological sucrose responsiveness

Unilateral stimulation of the tarsi with sucrose (Figure 2) elicited PER in 60–70% of the cases (Figures 2B–D). These values contrast with those usually obtained upon antennal stimulation with sucrose (90–100%). This disparity reflects the different sucrose sensitivities of these appendages (Marshall, 1935; De Brito Sanchez et al., 2008), which may be due to the fact that the tarsi are equipped with 15–30 times less receptors than the antennae (Whitehead and Larsen, 1976b).

The reciprocal experiment (Figures 3 and 4) showed that sucrose responsiveness was further decreased (PER was elicited in 20–40% of the cases) if a different substance was previously delivered to the tarsus opposite to that chosen for sucrose delivery. This decrease occurred irrespectively of the substance used as first tarsal stimulation. Water, for instance, induced the same decrease of sucrose responsiveness (Figure 3F) as quinine solution.

Experiments 1 and 2 suggest that responsiveness to sucrose in conditions of dual tarsal stimulation with sucrose and a different tastant depends on the primacy and nature of the tastant assayed. Sucrose solution delivered first elicits immediate PER which cannot then be inhibited by any other substance delivered afterwards on a different tarsus. In these conditions, sucrose acts as a “winner takes-all” stimulus. When a different tastant is first perceived by one tarsus, sucrose has no such effect when delivered to the other tarsus, thus showing that temporal primacy is also important. In this case, sucrose still elicits appetitive responses but these are significantly diminished, thus revealing an unspecific central inhibition. This indicates that a process of central integration takes place, probably at the level of the thoracic ganglion. Because different legs were used in these experiments, differences in responses can only be due to gustatory cross-comparison and inhibition between opposite tarsi (Dethier and Bowdan, 1992). In this process, two principles can be identified: (1) primacy is acquired by the first arriver and (2) asymmetries exist between sucrose and bitter/watery substances so that the former partially overcomes inhibition by the latter but not reciprocally. Further electrophysiological and behavioral experiments may help uncovering the mechanisms of this central inhibition.

Our electrophysiological recordings revealed that chaetic sensilla of the claws and of the tarsomeres differed in their response to sucrose solution 1 M. Tarsomere sensilla responded to sucrose solution in a similar way as to the contact electrolyte KCl (Figures 6A, 8A). Our attempt to establish a dose-response curve for sucrose in the case of tarsomere sensilla was not successful as there was no increase of cell responses with sucrose concentration over three orders of magnitude (Figure 7C). These results indicate that tarsomere sensilla do not host a sucrose receptor cell. On the contrary, claw sensilla responded 40–50 times more to sucrose 1 M than to KCl (Figure 8B). In these sensilla, temporal responses were always higher for sucrose than for other tastants assayed (Figure 9B). These results thus show that claw sensilla host a sucrose receptor cell and that the claws are the sucrose detecting region of the tarsi.

These conclusions were confirmed by the different responses obtained upon stimulation with mixtures of sucrose and bitter substances: while tarsomeres sensilla responded similarly to sucrose and to mixtures of sucrose and bitter substances (see Figure 8A), claw sensilla exhibited a significant reduction of responsiveness upon mixture stimulation (see Figure 8B). This reduction is consistent with the know inhibitory effect of bitter substances on sucrose receptor cells (Bernays and Chapman, 2000, 2001; De Brito Sanchez et al., 2005; Cocco and Glendinning, 2012).

### Behavioral and electrophysiological bitter responsiveness

Four substances that are perceived as bitter by humans—quinine, salicin, caffeine, and amygdalin—and that induce avoidance in the fruit fly (Meunier et al., 2003; Hiroi et al., 2004; Thorne et al., 2004; Wang et al., 2004; Marella et al., 2006; Masek and Scott, 2010) and in other insects (e.g., Chapman et al., 1991; Dethier and Bowdan, 1992; Ramaswamy et al., 1992; Bowdan, 1995) did neither inhibit appetitive responses in harnessed bees nor were detected via specific tarsal receptors. Different concentrations of these substances were unable to induce proboscis retraction once PER was elicited by sucrose (Figure 2). Two main explanations are possible: either peripheral detection of bitter taste is not possible via the tarsi, similarly to what happens with the antennae (De Brito Sanchez et al., 2005), or if it is possible, it is outweighed by sucrose stimulation so that the threshold for eliciting PER to sucrose would be lower than that for eliciting proboscis retraction to aversive substances.

Irrespectively of the concentration used, bitter substances delivered to one tarsus were unable to repress PER if sucrose was delivered to other fore tarsus, even if a lower level of PER was observed in these cases (Figures 3 and 4). Such decrease was induced not only by aversive substances but also by water, and was observable in bees which had their antennae intact or amputated. These results exclude both the lesion and the potential aversive nature of the first stimulation as a causal factor of the decrease in sucrose responsiveness. As mentioned above, the fact that sucrose responsiveness decreased when a different substance was first delivered in the leg opposite to the one used for sucrose stimulation speaks in favor of a central inhibitory process. Moreover, the fact that water yielded the same pattern of responses as concentrated quinine solution confirms that tarsal sensitivity for bitter substances is rather limited. Only, the lower level of PER induced by salicin upon sucrose stimulation compared to quinine solution and water (Figure 3F) indicates some inhibitory effect of salicin *per se*.

We were unable to detect specific electrophysiological responses to quinine, salicin, and amygdalin in electrophysiological recordings of chaetic sensilla located both on the tarsomeres and claws of the fore legs. The responses to bitter substances recorded at the level of tarsomere sensilla were, in fact, responses of a gustatory receptor cell triggered by the contact electrolyte KCl (0.1 mM). The fact that in these sensilla, no variations in responses to quinine were recorded over 3 orders of magnitude (Figure 7B) confirmed the absence of a cell tuned to this bitter substance in the tarsomeres.

The lack of an evident aversive effect of bitter substances is consistent with the report of von Frisch who mentioned that bitter solutions have no noticeable effect on the bees' ingestion of sugar solutions (Von Frisch, 1967). This may be due to the fact that sugars may mask bitter substances (Glendinning, 2002; Cocco and Glendinning, 2012). However, low concentrations of bitter substances such as phenolics and alkaloids in sucrose solution or nectar enhance food attractiveness while unnatural high concentrations of these substances diminish it (Hagler and Buchmann, 1993; Liu et al., 2004; Singaravelan et al., 2006; Liu et al., 2007). How are these differences detected in the light of an apparent absence of bitter receptors? Several explanations are possible: (1) such receptors exist but are neither located in the antennae (De Brito Sanchez et al., 2005) nor in the tarsi (this work); (2) responding aversively to bitter substances depends on the possibility of freely expressing avoidance; this is possible for free-flying bees but not for immobilized bees such as those used in our experiments (see discussion of this argument in Ayestaran et al., 2010); (3) detecting bitter components in nectar is possible via an indirect mechanism, namely the inhibition of sucrose receptors by bitter substances when these molecules appear together in a compound, such as in nectar with high levels of caffeine (Singaravelan et al., 2006). Inhibition of sucrose receptors by mixtures of either quinine and sucrose or amygdalin and sucrose was observed in claw sensilla (Figure 9B). This mechanism was also shown for sucrose receptors on the antennae of the honey bee (De Brito Sanchez et al., 2005) and for tarsal sucrose receptors of *Drosophila* (Meunier et al., 2003), blowflies (Dethier and Bowdan, 1989) and moth caterpillars (Bernays and Chapman, 2000, 2001), which are inhibited by several alkaloids and other substances that are bitter for humans.

### Electrophysiological salt responsiveness

Chaetic sensilla on the tarsomeres exhibited high responsiveness to NaCl 100 mM (Figure 6A). These sensilla thus host at least one receptor neuron highly responding to salts. This finding is in agreement with the well-documented preference of water foragers for salty water over pure water (Butler, 1940; Kiechle, 1961; Von Frisch, 1967). The reasons for such preference are unclear. It is assumed that bees prefer salty waters as they obtain from them minerals that are used for their own metabolism and for larval development (Dietz, 1971). But to our knowledge, no experiment has measured where and how bees do metabolize the salts collected during water foraging.

High sensitivity to saline solutions was also supported by the fact that tarsomere sensilla responded to extremely low KCl concentrations. In experiments 3 and 5, the contact electrolyte chosen was KCl 0.1 mM because higher concentrations of KCl (e.g., 10 mM) have been used in previous works as contact electrolyte to study gustatory responses in other appendages such as the antennae of bees and moths without inducing significant neural activity (De Brito Sanchez et al., 2005; Jørgensen et al., 2007). Yet, KCl 0.1 mM induced significant activity (Figures 5A, 6A). Tarsomere sensilla host at least a KCl receptor cell because spike frequency increased significantly with KCl concentration, especially in the range of low concentrations (Figure 7A). This increase is incompatible with the response of a water receptor cell and indicated that some responses to tastants were in fact responses to the diluted KCl used as contact electrolyte rather than to the tastants themselves. In *Drosophila melanogaster*, most taste sensilla house four gustatory receptor neurons, one of which is maximally sensitive to salt at low concentrations (L1 cell) (Meunier et al., 2003; Hiroi et al., 2004). The KCl cell reported in our work could therefore act as the equivalent of the L1 cell.

Contrarily to sucrose sensitivity, saline sensitivity was higher on the tarsi than on the antennae as shown by the difference of three orders of magnitude between the concentrations of KCl used as efficient contact electrolytes in either case [10 mM for the antennae (De Brito Sanchez et al., 2005) and 0.01 mM for the tarsi]. This difference may have an adaptive value: while sucrose is usually hidden in flower nectaries, thus requiring antennal assessment and not necessarily a direct contact with the tarsi, water surfaces are plain and are probably first contacted by the tarsi of a hovering bee to evaluate salt contents before making a decision on whether engaging or not in water collection.

## Conclusion

Our results provide an integrative view of gustatory tarsal detection in the honey bee, where the gustatory modality has received less attention than other modalities such as vision or olfaction (De Brito Sanchez, 2011) and where, in particular, the gustatory role of the tarsi has not been studied in detail.

Two main lines of future research emerge from our findings. On the one hand, it is imperative to provide a better characterization of molecular gustatory receptors present on single gustatory receptor cells hosted in gustatory sensilla of the tarsi. The genome of the honey bee (The Honeybee Genome Sequencing Consortium, 2006) has uncovered the presence of only 10 gustatory receptors genes (Grs; Robertson and Wanner, 2006) whose ligands are still unknown. It has been suggested that at least two of the bee Grs share homologies with the *Drosophila* Grs tuned to respond to sugars (Robertson and Wanner, 2006). Consistently with our findings, none of the bee Grs shows homologies with the different fly Grs specialized in bitter taste detection (Robertson and Wanner, 2006), which seems to be a general characteristic of Hymenoptera irrespective of their feeding style (e.g., ants; Bonasio et al., 2010). A fundamental task is therefore to identify the ligands and specificities of the Grs existing in the honey bee.

On the other hand, our results indicate that gustatory information from different legs is subjected to cross-comparison and can trigger, depending on which kind of taste is first perceived, a central excitatory state (sucrose 1st, non-sucrose 2nd) or a central inhibitory state (non-sucrose 1st, sucrose 2nd). This result raises the question of the mechanisms of central taste processing, for instance at the level of the thoracic ganglion where information from opposite tarsi converges. Further studies such as those performed in the thoracic ganglia of the locust (Newland et al., 2000a,b; Rogers and Newland, 2002), an insect that exhibits a taste sensitivity different from that of the honey bee given the presence of tarsal cell receptors tuned to bitter substances, should analyze how tarsal gustatory input is processed at the central level.

Finally, the fact that the taste modality has been relatively neglected compared to other modalities such as vision and olfaction, which have been intensively studied in the honey bee (Galizia et al., 2011), makes necessary a focus on taste perception through other body appendages besides the tarsi. As we have already characterized antennal taste perception (De Brito Sanchez et al., 2005), a next step is to provide an integrative study of taste perception at the level of the mouth parts. Contrarily to antennal and tarsal taste, for which previous studies were very scarce, taste via the mouth parts has been the subject of some detailed studies (Whitehead and Larsen, 1976a,b; Whitehead, 1978). Yet, the information obtained on antennal and tarsal taste receptors allows asking new questions. For instance, do antennal and tarsal taste receptors share the same gustatory logics with those of the mouth parts? Future work will allow answering this and other related questions.

### Conflict of interest statement

The authors declare that the research was conducted in the absence of any commercial or financial relationships that could be construed as a potential conflict of interest.
